# A phase I dose-escalation study to a predefined dose of a transforming growth factor-β1 monoclonal antibody (TβM1) in patients with metastatic cancer

**DOI:** 10.3892/ijo.2014.2679

**Published:** 2014-09-26

**Authors:** ALLEN COHN, MICHAEL M. LAHN, KRISTEN E. WILLIAMS, ANN L. CLEVERLY, CELINE PITOU, SUNIL K. KADAM, MARK W. FARMEN, DURISALA DESAIAH, ROBERT RAJU, PAUL CONKLING, DONALD RICHARDS

**Affiliations:** 1 Rocky Mountain Cancer Center - Midtown, Denver, CO, USA; 2 Eli Lilly and Company, Indianapolis, IN, USA; 3 Eli Lilly and Company, Erl Wood Manor, Windlesham, Surrey, UK; 4 Innovation Center Kettering Medical Center, Kettering, OH; 5 Virginia Oncology Associates, Norfolk, VA; 6 Tyler Cancer Center, Tyler, TX, USA

**Keywords:** metastatic cancer, monoclonal antibody inhibitor TβM1, pharmacodynamic and pharmacokinetic parameters, safety, transforming growth factor-β1

## Abstract

Transforming growth factor β (TGF-β) plays an important role in cancer. Monoclonal antibodies (mAb) designed to specifically block the TGF-β ligands, are expected to inhibit tumor progression in patients with metastatic cancer. TβM1 is a humanized mAb optimized for neutralizing activity against TGF-β1. The objective of this clinical trial was to assess the safety and tolerability of TβM1 in patients with metastatic cancer. In this phase I, uncontrolled, non-randomized, dose-escalation study, 18 eligible adult patients who had measurable disease per RECIST and a performance status of ≤2 on the ECOG scale were administered TβM1 intravenously over 10 min at doses of 20, 60, 120 and 240 mg on day 1 of each 28-day cycle. Safety was assessed by adverse events (as defined by CTCAE version 3.0) and possible relationship to study drug, dose-limiting toxicities and laboratory changes. Systemic drug exposure and pharmacodynamic (PD) parameters were assessed. TβM1 was safe when administered once monthly. The pharmacokinetic (PK) profile was consistent with a mAb with a mean elimination half-life approximately 9 days. Although anticipated changes in PD markers such as serum VEGF, bFGF and mRNA expression of SMAD7 were observed in whole-blood, suggesting activity of TβM1 on the targeted pathway, these changes were not consistent to represent a PD effect. Additionally, despite the presence of an activated TGF-β1 expression signature in patients’ whole blood, the short dosing duration did not translate into significant antitumor effect in the small number of patients investigated in this study

## Introduction

Transforming growth factor β (TGF-β) comprises 3 specific isoforms (-β1, -β2 and -β3) that are part of the TGF-β super family. These cytokines regulate diverse biological functions including cell proliferation, differentiation, motility, survival and apoptosis (1). The role of TGF-β in tumor biology is complex because it can act as both a tumor suppressor and a tumor promoter (2,3). TGF-β functions as a tumor suppressor by inhibiting cell growth in normal tissues, particularly in epithelial and lymphoid tissues (3). However, in the tumor cells it acts as an autocrine growth factor, creating an angiogenic, local immunosuppressive environment that enhances tumor growth and aggravates the invasive and metastatic tumor-cell behavior (4). The dual and opposing functions of TGF-β, including its ability to activate signaling molecules (5) other than the canonical SMAD pathway (6), has been implicated in the growth of a variety of human tumors, such as prostate, colon, breast, gastric, liver, renal and melanoma (7). Elevated plasma TGF-β1 concentration, the most prevalent TGF-β isoform in the systemic circulation in patients with invasive metastatic disease, are correlated with adverse outcomes (8–22).

Three approaches to inhibit the TGF-β pathway have been investigated, including the use of antisense oligonucleotides (ASOs), neutralizing monoclonal antibodies (mAb) against ligand-receptor interactions, and inhibitors of TGF-β receptor I kinases against the receptor-mediated signaling cascade. Though ASOs are highly specific inhibitors of TGF-β (23), they are limited by their organ and tissue penetration (24). The small-molecule kinase inhibitors (25,26), despite their advantages of high pathway selectivity and oral administration, are liable to cause toxicity and the cause of this toxicity is under investigation (27). The mAbs, designed specifically to block active TGF-β ligands and prevent their interaction with the type II receptor (28), are expected to inhibit tumor progression in patients with metastatic cancer (28). This concept has been tested in animal models using neutralization of TGF-β with mAbs (29,30). For example, the systemic and transgenic administration of the soluble TGF-β RII/Fc dimer that binds all 3 TGF-β isoforms, reduced the tumor burden as well as intravasation and metastasis in tumor animal models and in transgenic breast cancer mammary tumor virus (MMTV)-neu mice models, respectively (29).

TβM1 is a humanized mAb highly selective for neutralizing only active TGF-β1. Studies on the 4T1 Balb/c mice model demonstrated antitumor activity for both TGF-β1-specific mouse surrogate mAb and -neutralizing mAb 1D11 that neutralizes all 3 isoforms. The primary objective of this phase I clinical trial was to assess the safety and tolerability of TβM1 administered as monthly intravenous monotherapy in patients with metastatic cancer. The tested dose range of TβM1 was chosen to provide systemic TβM1 exposure predicted to have antitumor effects based on murine surrogate mAb data efficacy in a mouse model *in vivo*. Secondary objectives were to assess TβM1 systemic exposure and explore pharmacodynamic (PD) markers in whole blood by measuring global and specific changes in the expression of genes known to be associated with TGF-β pathway activation (29,31).

## Materials and methods

### Antibody

TβM1 is an IgG4 mAb with a preferential binding affinity to active TGF-β1. The *in vitro* ligand binding properties of TβM1 were determined using surface plasma resonance (SPR) to assess the binding specificity of the antibody to the 3 TGF-β ligands. TβM1 showed no binding to TGF-β2 and greater than 700-fold selectivity for TGF-β1 over TGF-β3.

### Dose selection

One rat PK/PD study was performed in 13762 (mammary carcinoma) syngeneic model with TβM1 at different dose levels. This was used to establish the EC_50_ value based on the SMAD2 phosphorylation in tumors. The choice of the doses was decided after a review of the preclinical package (rat PK/PD data with TβM1 and mouse efficacy data with the surrogate antibody), and animal toxicology data. The intravenous dose range of 20 to 240 mg was expected to be safe. Because TβM1 binds to active TGF-β1 at low concentrations, it was projected that doses of 120 and 240 mg would provide sufficient TGF-β1 blockade in cancer patients as assessed by systemic PD effects. Hence, the PD effects were expected to translate to clinical signals, such as tumor responses. This more focused approach for TGF-β inhibition may provide safety advantages over the non-selective-TGF-β mAb fresolimumab (32,33) which has produced antitumor responses in patients with melanoma and renal cell carcinoma (RCC) at similar doses.

### Study design

This was a phase I, multicenter open-label, uncontrolled, non-randomized, dose-escalation study of intravenously (IV) administered TβM1 in patients with metastatic cancer for whom no treatment of higher priority existed. At least 3 patients were enrolled in 1 of 4 cohorts receiving TβM1 flat doses of 20, 60, 120 and 240 mg, respectively, on day 1 of each 28-day cycle. Dose escalation to the next cohort proceeded only after 3 patients completed 1 treatment cycle without a dose-limiting toxicity (DLT) and after careful assessment of serum drug concentration and safety information. Hematologic or non-hematologic toxicity with a grade ≥3 was considered as a DLT in patients treated with the study medication at different dose levels according to the National Cancer Institute (NCI) and the Common Terminology Criteria for Adverse Events (CTCAE), version 3.0.

### Patients

Adult patients who provided written informed consent and had a histologic or cytologic diagnosis of cancer for which no proven effective therapy existed were included in the study. Eligible patients were required to have disease that was measurable or nonmeasurable as defined by the Response Evaluation Criteria in Solid Tumors (RECIST) and to have a performance status of ≤2 on the Eastern Cooperative Oncology Group (ECOG) scale. Patients were required to have adequate hematologic, hepatic, and renal functions and to have discontinued all previous therapies for cancer at least 4 weeks prior to study enrolment.

Exclusion criteria included medically uncontrolled cardiovascular illness, electrocardiogram anomalies, history of gastrointestinal (GI) bleeding, significant hemoptysis, hematuria within 3 months prior to study entry, serious pre-existing medical conditions (at the discretion of the investigator), unhealed wounds, history of autoimmune disease, symptomatic central nervous system (CNS) primary or metastatic malignancy, CNS active infection, human immunodeficiency virus (HIV), hepatitis, or immunosuppressive disease or hematological malignancies.

### Treatment

Lyophilized TβM1 at all doses (20, 60, 120 and 240 mg) was reconstituted in saline and administered as a 10-ml IV infusion via infusion pump at 10 ml per 10 min on day 1 of each 28-day cycle. Patients were monitored for any signs or symptoms of allergic reactions for at least 1 h after the administration of the study drug. No dose adjustments or reductions were allowed.

### Safety analysis

Safety was evaluated in patients who received at least one dose of TβM1. Safety assessment was based on the summaries of adverse events including severity (as defined by CTCAE version 3.0) and possible relationship to the study drug, DLTs and laboratory changes at each dose level. Safety was also analyzed by bone pain (level of pain and location) assessment, oral examination of the gingiva (to detect hyperplasia), skin assessment, evaluation of ECOG performance status, electrocardiogram (ECG), and echocardiography/Doppler. Clinically significant abnormal results were recorded as adverse events. Standard laboratory tests including chemistry, hematology and urinalysis panels were also performed. All concomitant medications were documented throughout the patient’s participation in the study.

### Efficacy analysis

Data on any clinical benefit and tumor response were tabulated. No formal efficacy analysis was performed.

### Bioanalytical methods

A validated enzyme-linked immunosorbent assay (ELISA) method (ALTA Analytical Laboratory, San Diego, CA, USA) was used to analyze the human serum samples for TβM1. The lower and upper limits of quantification were 7.5 and 90.0 ng/ml, respectively. In order to yield results within the calibrated range, samples above the limit of quantification were diluted and reanalyzed. During validation, the inter-assay accuracy (% relative error) ranged from −13.5% to 2.0% while the inter-assay precision (% relative standard deviation) ranged from 12.5 to 13.4%. TβM1 was stable for up to 365 days when stored at approximately −70°C.

### Pharmacokinetic (PK) methods

All patients who received at least one dose of TβM1 and had serum samples collected were subject to pharmacokinetic analyses. The PK parameters, area under the concentration-time curve (AUC) and half-life for TβM1 were computed by standard noncompartmental methods of analysis using Win Nonlin Professional Edition (version 5.3) on a computer that met or exceeded the minimum system requirements for this program with appropriate and validated software. The extent of dose proportionality was assessed using estimated AUC. AUC estimates were log-transformed prior to analysis and ratios of dose-normalized geometric means and the corresponding 90% confidence intervals were provided.

### Pharmacodynamic methods

Pharmacodynamic assessments included quantitative reverse transcriptase-polymerase chain reaction (RT-PCR) assays, multianalyte immunoassay panels (MAIP) [Rules-based Medicine (Myriad RBM), Austin, TX, USA], and gene expression microarrays (Affymetrix^®^, Santa Clara, CA, USA). Blood for serum collection was collected prior to initiation of treatment and at various times for up to 12 days following treatment. Normal blood collected from five healthy volunteers was used for bioanalytical comparison of microarray profiles between patients with disease and healthy subjects. This collection of normal blood samples was not part of the current study and was part of a previous publication (34). Additionally, CD4^+^CD25^+^ T-cell counts and total T cells for the lymphocyte population were monitored to evaluate immune function by standard flow cytometry. All patients undergoing PD assessments who yielded data from RT-PCR, MAIP and gene expression microarrays were included in the analyses.

### RT-PCR assay of gene expression

Measurements observed with repeated qRT-PCR normalized to glyceraldehyde-3-phosphate dehydrogenase, housekeeping gene, were recorded for SMAD7, TMEPAI, OCIAD2 and CA1 and analyzed using a linear mixed model since the normality assumption was appropriate. Dose, nominal time point, and dose-by-time-point interaction were included as fixed effects, baseline (predose) value as covariate, and subject as random effect in the model, which allowed the formal pre-post dosing comparison of the gene expression. Similarly, observed percentage changes from baseline (predose) of postdose qRT-PCR measurements were analyzed with dose, nominal time point, and dose-by-time-point interaction as fixed effects, screening value as covariate, and subject as random effect, and derived model percentage changes plotted against time. The assumed covariance structure for both models was compound symmetry, which was deemed more appropriate for the data, while the degrees of freedom for the tests of fixed effects were calculated using the Kenward and Roger method.

### Multianalyte immunoassay panel (MAIP)

The MAIP repeated measurements data from 89 analytes were plotted over time by dose groups to which logarithmic transformation was applied, as appropriate to the data.

### Affymetrix analysis

Gene-expression profiling using Affymetrix U133 microarray data was analyzed. Previously, evaluation of the effects of TGF-β1 with cell-lines and isolated normal peripheral blood mononuclear cells (PBMCs) revealed that the difference between TGF-β1 stimulated and unstimulated conditions was best captured by 8 genes from the array: SMAD7, CRYBB1, ATF3, TFDP2, CA1, OCIAD2, TMEPAI, and TMCC2, GAPDH was used as a normalizer. A linear mixed model with random patient effect was used to analyze the change from pre-TβM1 treatment baseline in the repeated expression measures (log scale). Nominal time point and *ex vivo*-based prediction of TGF-β1 stimulated versus TGF-β1 unstimulated at baseline were treated as fixed effects. The model LS means and p-values at nominal time points were reported. Additionally, we evaluated an extended set of 37 genes previously shown to be associated with TGF-β1 pathway activation in the *ex vivo* PBMC stimulation and expression measurement assay (35). Signal values for the probe sets corresponding to the 37 genes were normalized and represented as an expression index according to Zhaou and Rocke (36).

## Results

### Patient disposition

A total of 18 patients entered the study and received at least 1 dose of TβM1. The majority of patients were treated for 2 cycles (n=14). Among the reasons for discontinuation, the most common reason was progressive disease (n=16). One patient died from his bladder cancer during the study and 1 patient discontinued per own decision.

The second dose cohort (60 mg) was expanded to a total of 8 patients due to a grade 3 diarrhea DLT in one of the initial 3 participants. Following confirmation that there were no additional DLTs and assessment of the systemic exposure and safety information, the study continued with escalation up to the predefined 240-mg dose.

### Patient demographics, disease characteristics and disposition

The patient baseline demographics by TβM1 dose are described in Table I. The mean age of the patient population was 62 years. Female patients comprised 50% (n=9) of the study population. A majority of study population was Caucasian (n=17, 94%). In general, the baseline demographics were similar between the dose groups.

All 18 patients who entered the study had an ECOG performance status ≤2, with the majority of patients (n=15, 83%) having a score of 1 (Table I). Six patients (33%) had an initial pathological diagnosis of adenocarcinoma of colon, 3 (17%) had adenocarcinoma of rectum, and 2 (11%) had not-otherwise-specified sarcoma. The 7 remaining patients (39%) each had unique cancer diagnoses.

### Safety measures

Table II describes the AEs, TEAEs, CTCAE grade toxicity, SAEs and discontinuations. One patient with bladder cancer died during the study due to progressive disease and 8 reported at least 1 SAE. Two of the 8 patients (11%) with an SAE had, in the investigator’s opinion, an SAE possibly related to the study drug. None of the patients discontinued due to adverse events. Nine patients (50%) had at least one TEAE that in the opinion of the investigators was treatment related. Regardless of CTC grade, fatigue, nausea and diarrhea were the most frequently reported possibly drug-related TEAEs. Each of these events occurred in 3 patients, which corresponds to 17% of the treated patients.

Thirteen patients (72%) reported at least 1 grade 3/4 CTCAE toxicity (Table II). The majority of possibly drug-related TEAEs were assessed as grade 1 or 2. Two grade 3 toxicities related to the study drug were reported including diarrhea (n=1 at 60 mg in cycle 1) and generalized muscle weakness (n=1 at 240 mg in cycle 1). No grade 4 toxicities related to the study drug were reported. The grade 3 diarrhea was both an SAE and a DLT that ultimately led to the expansion of the 60-mg group (cohort 2). The event resolved and the patient remained on the study until later discontinuation due to progressive disease. The grade 3 muscle weakness was also an SAE and the event had not resolved when the patient discontinued from the study due to patient decision and was placed in hospice care. In addition, 1 patient at the 240-mg dose level (cohort 4) had a treatment-emergent grade 2 CTCAE laboratory abnormality (low hemoglobin) that was considered, in the opinion of investigator, as a possibly study-drug-related.

There were no changes reported in vital signs with regard to temperature, heart rate, or blood pressure after administration of TβM1 in any of the patients on therapy and by dose. No clinically relevant changes in ECGs were observed.

Other safety measures determined were: skin assessments, oral examinations and bone-pain assessments. One drug-related dry mouth was reported and 1 related TEAE was reported for each including intermittent lip peeling, facial ulceration and rash. All events resolved during the study.

### Pharmacokinetic measures

The systemic exposure after IV administration of TβM1 is presented in Table III across 20 to 240 mg dose ranges (cohorts 1 to 4). The terminal half-life (t_1/2_) remained relatively constant (at approximately 9 days) and ranged from approximately 5.67 to 15.38 days. Following a 10-min infusion, TβM1 AUC increased with dose. Table IV presents the results of the statistical analysis of dose proportionality for AUC from zero extrapolated to infinity (AUC_0-∞_ in cycle 1) using the power model for TβM1.

### Pharmacodynamic measures

**Figure 1:**
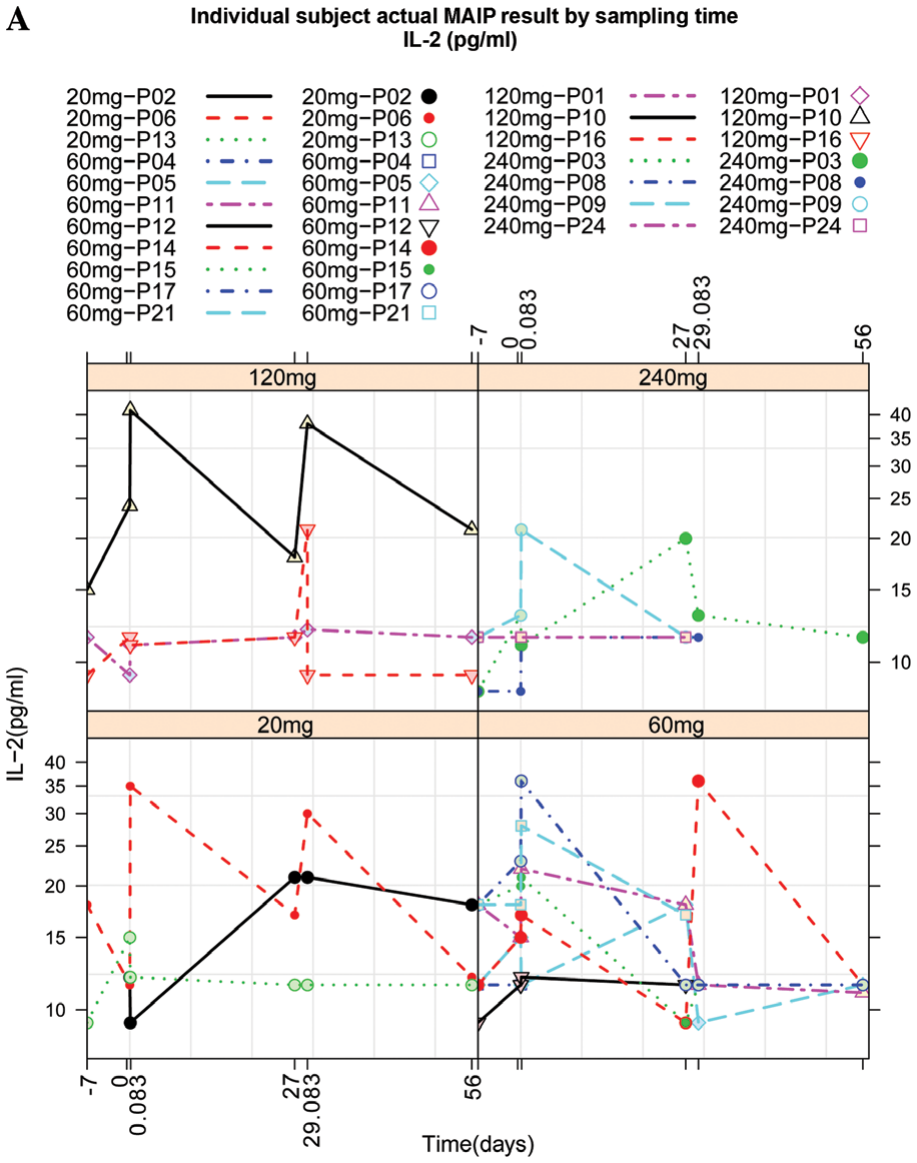

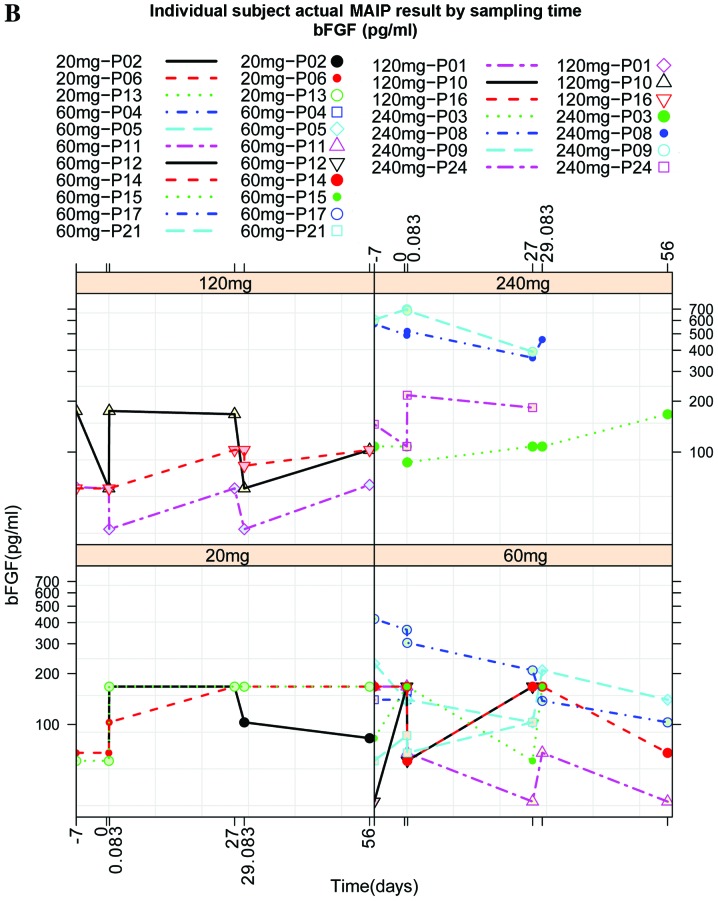

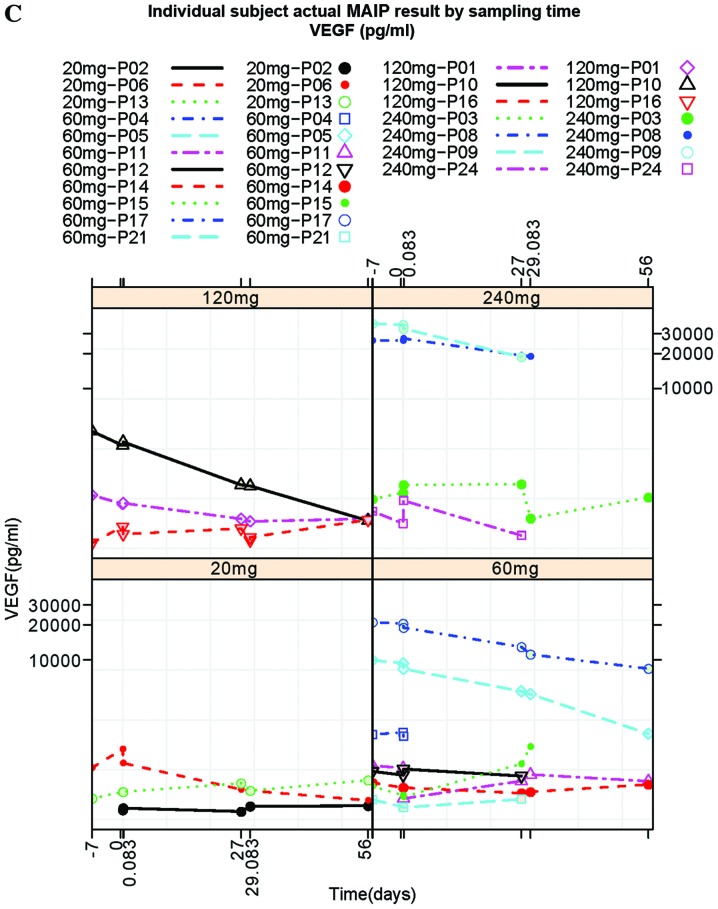
Individual patient profiles of multianalyte immunoassay panel test results over sampling time points. (A) Levels of IL-2 at different doses (20, 60, 120 and 240 mg) of TβM1. The levels of these biomarkers were measured at different time points after administration as shown on X-axis. Time point 1, ≤7 days prior to first dose; time point 2, cycle 1, predose; time point 3, cycle 1, 2 h post-dose; time point 4, cycle 1, 27th day; time point 5, cycle 2, 2 h post-dose; time point 6, cycle 2, 27th day. (B) Levels of bFGF at different doses (20, 60, 120 and 240 mg) of TβM1. The levels of these biomarkers were measured at different time points after administration as shown on X-axis. Time point 1, ≤7 days prior to first dose; time point 2, cycle 1, predose; time point 3, cycle 1, 2 h post-dose; time point 4, cycle 1, 27th day; time point 5, cycle 2, 2 h post-dose; time point 6, cycle 2, 27th day. (C) Levels of VEGF at different doses (20, 60, 120 and 240 mg) of TβM1. The levels of these biomarkers were measured at different time points after administration as shown on X-axis. Time point 1, ≤7 days prior to first dose; time point 2, cycle 1, predose; time point 3, cycle 1, 2 h post-dose; time point 4, cycle 1, 27th day; time point 5, cycle 2, 2 h post-dose; time point 6, cycle 2, 27th day. IL-2, interleukin-2; bFGF, basic fibroblast growth factor; VEGF, vascular endothelial growth factor.

While IL-2 levels were increased approximately 2 h after IV administration of TβM1, other plasma protein were decreased or not changed after dosing of TβM1 (Fig. 1A–C). Vascular endothelial growth factor (VEGF) levels were decreased at the 60-mg and 240-mg doses of TβM1 and basic fibroblast growth factor (bFGF) levels were also reduced at 60-mg and 240-mg doses of TβM1. These effects were seen consistently in patient cohorts.

**Figure 2:**
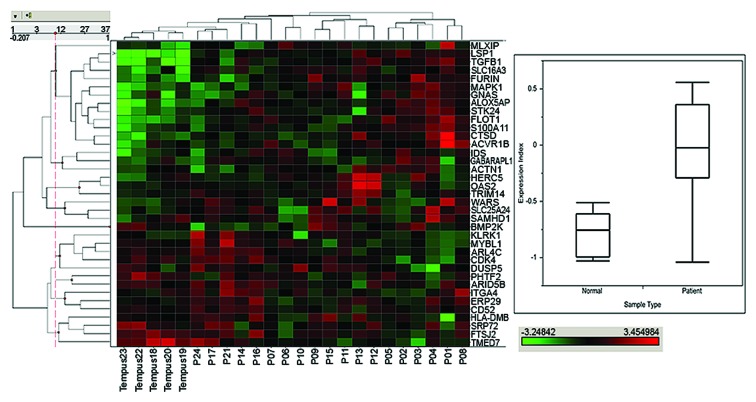
Hierarchical clustered heat map representation of the expression signals from 37 genes associated with systemic TGF-β1 pathway activation (study samples are designated with an underlying blue bar). Each column represents expression signals for an individual sample for genes designated to the right. Samples designated as Tempus with an underlying green bar were from normal subjects and were used for bioanalytical comparison (samples collected from healthy subject in a previously published study). The adjacent box plot is a representation of the 37-gene calculated expression index values for normal (Tempus) samples and patient samples. The upper and lower lines of the box represent the 75th and 25th percentile and the line within the box represents the median value. The outward whiskers represent the upper and lower ranges.

**Figure 3:**
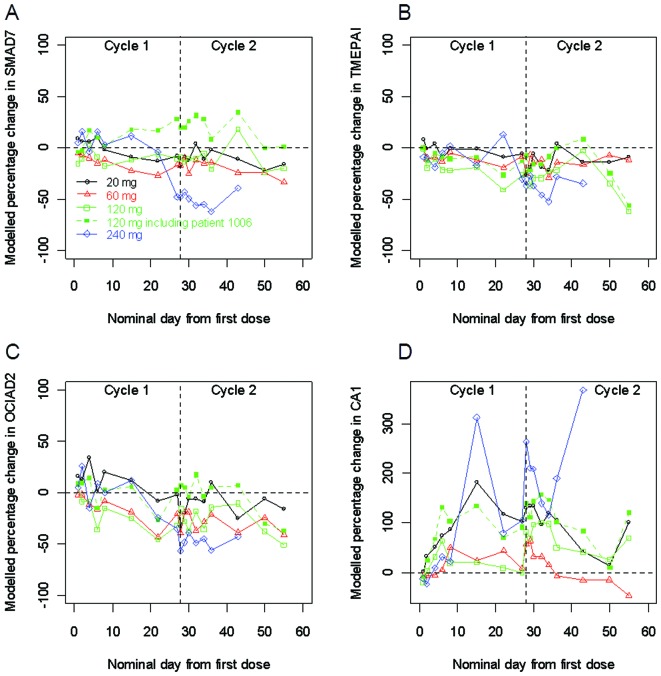
Change in the expression of genes as a function of dose in patient whole blood as measured by qRT-PCR analysis for TβM1 administered over cycle 1 and cycle 2 (20, 60, 120 and 240 mg). (A) SMAD7; (B) TMEPA1; (C) OCIAD2 and (D) CA1.

Changes in expression of genes associated with the TGF-β1 pathway activation have been reported to influence tumorigenesis in genetically altered animals (37). We previously observed (35) changes in global gene expression and associated a 37-gene whole blood expression profile that was associated with markers of systemic TGF-β1-dependent pathway activation. Here, we show the measurement of whole blood expression signals for the 37 genes and their association with TGF-β1 pathway activation status compared with whole blood from normal subjects (Fig. 2). A hierarchically clustered heat map of the corresponding probe set signals in pretreatment patient whole blood clearly separated certain patient samples from normal samples collected in Tempus tubes. In normal samples designated Tempus 18–23 (upper left region of left panel) there is a region where several genes are expressed at lower level (green) in normal subjects compared with increasing expression (going from green to red) in patients. Similarly there is a set of genes in the lower left where expression in patient samples is higher than in normal subjects. The median expression index of the study samples from both populations of subjects represented in the box and whisker plot (Fig. 2, right panel) was almost 2-fold higher in cancer patients than normal subjects, suggesting increased systemic TGF-β1 activation status in patients. In each case, the area in the box represents the upper and lower 75th and 25th percentile and the line within it represent the median. The upper and lower maximal values are represented by the outward directional extensions. Taken together, these data show that in cancer patients, there is a trend where the whole blood expression, represented by the calculated expression index, is shifted toward a more activated TGF-β1 system. We also measured the expression of selected genes known to be regulated by TGF-β1 exposure to cultured cells and/or *ex vivo* peripheral blood mononuclear cells (PBMCs). Fig. 3 depicts the expression of selected genes using qRT-PCR. Of the selected genes, SMAD7, TMEPAI and OCIAD2 were normally upregulated *in vitro* with TGF-β1 addition while CA1 was downregulated (data not shown). The expression of CA1 increased after TβM1 treatment, especially at the 240-mg dose (panel D). The gene expression measurement of SMAD7 and TMEPAI, both known to be negative regulators of TGF-β1 activation, were generally negative in post-treatment especially at the highest dose, indicating reduced TGF-β1 activation and pointing to a possible PD effect with TβM1 treatment (panels A and B). Additionally, the level of OCIAD2 expression in whole blood appeared to be reduced upon treatment at all doses (panel C). Despite hints of a PD effect, the changes noted were not consistent across treatment groups.

### Clinical efficacy measures

Clinical efficacy was a secondary objective in this study. Of 18 patients who received at least 1 dose of TβM1, 13 had at least 1 postdose for tumor response. Based on RECIST response assessment, the best overall study response was stable disease for 7 patients and progressive disease for 6 patients. The tumor markers in all patients, including lactate dehydrogenase (LDH), increased or were only briefly reduced (less than 1 cycle).

## Discussion

TGF-β inhibitors target a complex biology in cancer. Patients with advanced or metastatic conditions seem to have particularly high tumor expression, or produce large amounts of TGF-β ligands (17). Although inhibiting only one isoform may not be sufficient to achieve antitumor efficacy, we hypothesized that targeting TGF-β1 would be sufficient to obtain tumor responses, because it is the most prevalent ligand in plasma or serum of patients with invasive metastatic disease and correlates with adverse outcomes (8–22).

The present study was designed to evaluate the safety, PK and PD effects of TβM1 at a prespecified dose range in patients with advanced metastatic cancer. In contrast to typical phase I dose-escalation studies in oncology, the objective of this study was not to investigate a maximum tolerated dose (MTD). Given that TβM1 is hypothesized to bind and neutralize TGF-β1 at low concentrations, it was proposed that the consequence of blocking TGF-β1 would result in significant PD effects in cancer patients at monthly intravenous doses of 120 to 240 mg. The PD effects were expected to translate to clinical signals such as tumor responses, because a neutralizing mAb with a lower affinity to all three TGF-β ligands (32,33) produced antitumor responses in patients with melanoma and renal cell carcinoma (RCC) at similar doses.

### Safety

Overall, TβM1 was well tolerated across the 20 to 240 mg dose range. No pattern of a dose-response relationship with AEs was noted and no patient discontinued due to AEs or SAEs. Sixteen of the 18 cancer patients discontinued study treatment due to progressive disease. The only death was a patient in the 60-mg cohort who died due to his bladder cancer. One patient who received 240 mg discontinued study treatment per own decision. Except for the escalation from the first to second dose cohort (20 and 60 mg, respectively), dose escalation of TβM1 preceded as planned and the only DLT identified was a self-limited grade 3 diarrhea observed in one of the initial 3 patients in the 60-mg cohort. Logistic regression analysis for the probability of experiencing a DLT was not performed, as there was only one occurrence of a DLT.

### Pharmacokinetic profile of TβM1

The PK profile is consistent with other known monoclonal antibodies (38). Since this agent is an IgG4 and is given IV, these study data provide additional information on the PK behavior of this class of mAb compared with the IgG1 and IgG2 backbone. TβM1 measurements of anti-drug antibody (ADA) confirmed that less than 1% of the patients developed antibodies against TβM1 (one patient in this study).

### Pharmacodynamics of TβM1

The small sample size limits assessments of PD effects at individual doses. Regarding the specific gene expression panel that was previously identified to be regulated by TGF-β inhibition (39), there was non-significant reduction of SMAD7, TMEPAI and OCIAD2 at the 240-mg dose. Significant effects might have occurred with a larger sample size, more frequent dosing or higher doses. This would suggest that the dose of TβM1 was not effective enough in blocking TGF-β1 levels in humans to achieve a reduction in gene expression in PBMCs. Notably, there was no dose-related decrease; rather the reduction for all dose levels was similar. Hence, it is also possible that the observations for the 240-mg dose level were chance events. The comprehensive gene expression profiling also suggested that the doses were not sufficient to decrease the TGF-β1-associated signaling as determined by the *STK24* gene expression. After TβM1 administration, VEGF and bFGF were reduced in some patients as detected by the MAIP. While this indicates a possible TβM1 treatment effect, other markers of tumor progression were increased in the same and other patients. For instance, IL-6 was increased in a patient with RCC and IL-8 was increased in 5 patients (data not shown). In addition to these pro-inflammatory markers, PAI-1 and TIMP-1 were increased (data not shown). The MAIP detects tumor markers which can be compared to standard chemistry tests. For instance, the carcinoembryonic antigen (CEA) values obtained by standard serum chemistry were correlated with the elevated CEA values detected by the MAIP. Unfortunately, as already shown in some patients with standard tumor marker evaluation, all the MAIP-based tumor markers increased during treatment with TβM1. Overall, the MAIP panel depicts a situation that is consistent with tumor growth and not with tumor response and in some instances is expected based on previous evaluations (40).

### Clinical response of TβM1

The best clinical response in this study was stable disease. There were 4 patients who received 3 cycles and only 1 patient who was treated for 4 cycles. While this result is consistent with the benefits observed in general phase I oncology patients (41), the results on balance do not favor further clinical development of TβM1 as a treatment for non-specific types of cancer. The tumor markers in all patients, including lactate dehydrogenase levels, increased or were only briefly reduced (less than 1 cycle). All this suggests that the TGF-β1 blockade by the dosing regimens employed was either inadequate, that TGF-β1 is not sufficiently active in tumor growth, or that its inhibition leads to activation of other pro-growth pathways in patients where the relevance of TGF-β-dependent growth has not been predetermined at study entry. Compared with the fresolimumab (GC-1008) clinical observation, another IgG4 mAb but directed against all 3 ligands (32), it is possible that TβM1 may have been active in patients with melanoma. However, no melanoma patients were included in this study.

In summary, TβM1 is safe when administered once monthly by IV for 10 min. No MTD was observed. Reduction in PD marker levels of VEGF and bFGF suggests minor activity of TβM1 on the targeted pathway at the dose regimens investigated. PD effects on gene expression profiles do not translate into significant antitumor effects in patients. This lack of a consistent PD response and a clinical antitumor effect in the various cancers included in this trial failed to identify a tumor type responsive to isolated TGF-β1 suppression that warrants further study.

## Figures and Tables

**Table I:** Patient demographics and disease characteristics of all enrolled and treated patients by dose.

	Part A				
	
Parameter	20 mg, N=3	60 mg, N=8	120 mg, N=3	240 mg, N=4	Total, N=18
Age, years					
Mean (SD)	73 (7.6)	61 (20.7)	61 (12.1)	57 (15.5)	62 (16.5)
Median (range)	75 (65, 80)	71 (23, 81)	65 (47, 70)	53 (43, 79)	68 (23, 81)
Gender, n (%)					
Female	2 (67)	4 (50)	1 (33)	2 (50)	9 (50)
Male	1 (33)	4 (50)	2 (67)	2 (50)	9 (50)
Race, n (%)					
Caucasian	3 (100)	8 (100)	2 (67)	4 (100)	17 (94)
African American	0 (0)	0 (0)	1 (33)	0 (0)	1 (6)
ECOG PS, n (%)					
0	0 (0)	1 (13)	1 (33)	1 (25)	3 (17)
1	3 (100)	7 (88)	2 (67)	3 (75)	15 (83)
Basis of initial pathological diagnosis, n (%)					
Histopathological	3 (100)	7 (88)	3 (100)	4 (100)	17 (94)
Cytological	0 (0)	1 (13)	0 (0)	0 (0)	1 (6)
Initial pathological diagnosis, n (%)					
Adenocarcinoma, colon	2 (67)	2 (25)	2 (67)	0 (0)	6 (33)
Adenocarcinoma, rectum	0 (0)	1 (13)	1 (33)	1 (25)	3 (17)
Sarcoma, NOS	1 (33)	1 (13)	0 (0)	0 (0)	2 (11)
Other^a^	0 (0)	4 (50)	0 (0)	3 (75)	7 (39)

a Others include 1 each are: adenocarcinoma of gastric, adenocarcinoma of pancreas, carcinoma of breast, carcinoma of renal cell, carcinoma of cervix squamous cell, carcinoma of lung squamous cell, carcinoma of urothelial transitional cell.

N, total enrolled and treated population; n, number of patients in the group; SD, standard deviation; ECOG PS, Eastern Cooperative Oncology Group Performance Status; NOS, not otherwise specified.

**Table II:** Summary of adverse events in all enrolled and treated patients by dose.

	20 mg, N=3 n (%)	60 mg, N=8 n (%)	120 mg, N=3 n (%)	240 mg, N=4 n (%)	Total, N=18 n (%)
Patients with ≥1 AE	3 (100)	8 (100)	3 (100)	4 (100)	18 (100)
Possibly related to study drug	2 (67)	4 (50)	1 (33)	2 (50)	9 (50)
Patients with ≥1 TEAE	3 (100)	8 (100)	3 (100)	4 (100)	18 (100)
Possibly related to study drug	2 (67)	4 (50)	1 (33)	2 (50)	9 (50)
Patients with ≥1 Grade 3/4 CTCAE	1 (33)	8 (100)	0 (0)	4 (100)	13 (72)
Possibly related to study drug	0 (0)	1 (13)	0 (0)	1 (25)	2 (11)
Patients with ≥1 SAE	0 (0)	6 (75)	0 (0)	2 (50)	8 (44)
Possibly related to study drug	0 (0)	1 (13)	0 (0)	1 (25)	2 (11)
Patients who discontinued due to AE	0 (0)	0 (0)	0 (0)	0 (0)	0 (0)
Patients who discontinued due to SAE	0 (0)	0 (0)	0 (0)	0 (0)	0 (0)

AE, adverse event; N, total safety population size; n, number of patients with at least one adverse event per event type; SAE, serious adverse event; TEAE, treatment-emergent adverse event.

**Table III:** Pharmacokinetics parameters per dose group and per cycle reported as median (range) or geomean and CV% when n >3.

	20 mg		60 mg			120 mg				240 mg	
				
Parameter	Cycle 1 N=3	Cycle 2 N=3	Cycle 1 N=8	Cycle 2 N=4	Cycle 3 N=1	Cycle 1 N=3	Cycle 2 N=3	Cycle 3 N=1	Cycle 4 N=1	Cycle 1 N=4	Cycle 2 N=2
T_1/2_, (day)	10.50 (6.30–14.00)	11.50 (7.63–13.40)	7.63 (40.51)	8.21 (18.02)	10.04	8.46 (50.64)	8.88 (22.30)	11.90	15.38	7.33 (8.23)	5.67 (4.83–6.54)
AUC_0-∞_ (μg·h/ml)	1,490 (468–1,722)	1,313 (446–2,004)	2,922 (43.80)	3,303 (42.90)	2,656	5,117 (33.60)	5,061 (21.70)	4,889	6,707	8,987 (31.00)	8,118 (6,025–10,210)

a Due to a less intensive sampling time, the values should not be compared with cycles 1 and 2. AUC_0-∞_, area under the concentration-time curve from zero extrapolated to infinity.

**Table IV:** Statistical analysis of dose proportionality for AUC_0-∞_.

PK variable	Doses ratio	Predicted geometric mean PK parameter values	R_dnm_ 90% confidence interval
AUC_0-∞_	12	1,112 to 9,232	0.69 (0.41, 1.17)
	6		0.77 (0.52, 1.12)
	4		0.81 (0.61, 1.09)
	2		0.90 (0.78, 1.04)

AUC_0-∞_, area under the concentration-time curve from zero extrapolated to infinity; Cmax, maximum plasma concentration; PK, pharmacokinetics; R_dnm_, ratio of model-predicted mean values for high and low dose, normalized for dose.
